# Laser Forced Dehydration of Benign Vascular Lesions of the Oral Cavity: A Valid Alternative to Surgical Techniques

**DOI:** 10.3390/medicina60050822

**Published:** 2024-05-16

**Authors:** Margherita Gobbo, Luca Guarda-Nardini

**Affiliations:** Unit of Oral and Maxillofacial Surgery, Ca’ Foncello Hospital, Piazzale Ospedale, 1, 31100 Treviso, Italy; luca.guardanardini@aulss2.veneto.it

**Keywords:** vascular malformation, diode laser, angioma, blue light, conservative

## Abstract

*Background and Objectives*: Low-flow vascular lesions are commonly encountered in the oral cavity and may require removal due to aesthetic concerns, repeated bleeding or a cluttering sensation. Laser devices represent an excellent aid due to their affinity with blood and to their biostimulating properties and have been substituting traditional excision in selected cases. *Materials and Methods*: In this study, 30 patients presenting with low-flow oral vascular lesions were included. The lesions were clinically evaluated as follows: lesion’s site, reason for treatment, lesion’s dimensions, confirmation of positive diascopy via compression with a glass slide and photograph. The lesions were treated with laser forced dehydration (LFD) and then followed-up after 3 weeks, 6 months and 1 year. The laser source was a K-Laser Blu Derma (Eltech, K-Laser S.r.l., Via Castagnole, 20/H, Treviso, Italy). In the case of incomplete healing, a further protocol was performed at the three-week follow-up, and a further follow-up was scheduled for three weeks after. The following aspects were evaluated at each appointment: pain, using a Numeric Rating Scale (NRS) from 0 to 10 (0 = no pain, 10 = worst pain ever); the need to take painkillers (day of intervention and during follow-up); bleeding (yes/no); scar formation. *Results*: Complete regression was obtained in all patients, with no side effects. Only one patient required a second LFD protocol. NRS was 0 for all patients for the whole duration of the follow-up. None of the patients took painkillers on the day of the intervention and during the follow-up. One patient declared slight bleeding the day of the intervention, which she easily managed at home. One patient showed a small non-retracting and non-painful scar at the three-week follow-up. No recurrences were found after six months and one year. *Conclusions*: LFD targets endogenous chromophores, minimizing damage to adjacent tissue and limiting side effects. LFD is effective and could be considered a conservative alternative to traditional excision in low-flow lesions.

## 1. Introduction

Among vascular anomalies, vascular malformations (VMs) are classified based on clinical, histological and histochemical features and are characterized by blood vessel abnormalities. They are distinguished from vascular tumors, which are characterized by endothelial proliferation and aggressive behavior, instead. Among VMs, according to the International Society for the Study of Vascular Anomalies (ISSVA) [1] classification, lesions may be (1) simple, (2) combined, (3) of major named vessels or (4) associated with other lesions (mainly syndromes). Simple lesions include the following: capillary malformations, lymphatic malformations, venous malformations, arteriovenous malformations and arteriovenous fistula [2]. A low-flow lesion originates from a capillary, venous or lymphatic vessel or from a combination of vessels. Malformations with an arterial component (most commonly arteriovenous) are considered high-flow lesions. Simple VMs are low-flow lesions and are frequently encountered in the oral and maxillofacial regions. Despite being asymptomatic, they often require treatment due to aesthetic concern, recurrent bleeding—occurring after accidental or chewing trauma—and cluttering sensation due to their position or increasing size [3].

There is no universally accepted treatment for VMs in the oral cavity, despite several algorithms having been proposed [4]. Among the treatment possibilities, sclerotherapy with agents, like 3% tetradecyl sulphate or ethanol injected into the lesion, lead to disruption of endothelial cells and, consequently, luminal obliteration, fibrosis and shrinkage of the lesion. Cryotherapy and irradiation are described alternatives [5]. Steroids injected intralesionally may lead to an 84% response, also in the case of large lesions, but are not free of side effects, like fat atrophy, local necrosis, thickness and calcification. Surgery is generally reserved for lesions that are refractory to less-invasive treatments [4].

In recent years, the coagulative properties of several laser devices have promoted their use as a conservative and effective technique [6]. A technique called “laser forced dehydration” (LFD) can be obtained by moving the beam on the lesion in touchless modality without lingering on the same area for more than 1–2 s, in order to avoid thermal damage and overheating. During enlightening, the lesion should visibly change color, from bluish red to white. The process is mediated by the high absorption and affinity of blue light with hemoglobin, the key processes of LFD [7]. LFD does not need any anesthesia and can be repeated in case of failure. Moreover, it has a reduced risk of bleeding and an enhanced healing capacity, thanks to the delivery of a laser light to the tissue.

The aim of this study was to treat patients affected by low-flow VMs of the lips or oral cavity by LFD using a blue-light diode laser and to clinically follow-up the participants over a one-year period.

## 2. Materials and Methods

The present study was conducted between March 2022 and March 2024 at the Unit of Oral and Maxillofacial Surgery of “Ospedale Ca’ Foncello” (Treviso, Italy). Ethical approval was obtained (Nucleo Ricerca Clinica, Treviso, prot. 73512 (19 April 2021) studio osservazionale 976/CE), and all the included subjects signed written informed consent.

Benign simple capillary or venous malformations, according to the ISSVA classification [1], were treated using LFD. Inclusion criteria were pigmented black or bluish, soft, non-pulsatile lesions, which showed positive diascopy, involving any area of the oral cavity, which was reachable with the laser device. Exclusion criteria were patients younger than 18 years old, pregnant or breastfeeding women, patients not willing to perform a one-year follow-up. Laser source was K-Laser Blu Derma (Eltech, K-Laser S.r.l., via Castagnole 20/H, Treviso, Italy).

In the present study, 30 patients affected by low-flow VM of the oral cavity were treated with LFD using a blue-light diode laser and the so-called “Leopard Technique”, a multiple-spot pulsed irradiation technique that spares the epithelium and promotes smooth healing [8]. Specifically, each lesion was irradiated interposing a glass transparent slide between the epithelium and the laser beam, in order both to protect the lesion from excessive heating—and, thus, limiting unwanted effects like bleeding or ulceration—and to compress the lesion and deliver the beam to its core [8].

### 2.1. Study Protocol: Clinical Evaluation

The lesions were clinically evaluated, and the following data were recorded: lesion site, reason for treatment (bleeding, aesthetic, cluttering), lesion dimensions (in millimeters, using a calibrated paper ruler and considering the wider diameter as final measure), confirmation of positive diascopy via compression with a glass slide, photograph of the lesion.

### 2.2. Study Protocol: Laser Application

No anesthesia was needed. The lesion was treated with the following protocol, interposing a compressing glass slide between the lesion and the laser beam:

(1) Plain angioma (named after the default program in the device): using a red tip (code MP387 C), 445nm and 970 nm wavelength, 164 mJ energy, 3.28 Hz frequency, duty cycle 2%, 3 mm working distance, directly furnished by the red tip.
(2) Ruby angioma (named after the default program in the device): using a silver tip (code MP387 A), 445 nm and 970 nm wavelength, 909 mJ energy, 22.73 Hz frequency, duty cycle 9%, 2–3 mm working distance established by the operator.

The first protocol is less energetic, helps reduce the risk of bleeding and is more controlled, since the tip employed gives a pre-determined working distance. The second protocol is more energetic, and the position of the beam should be decided by the operator, so it has a sharper learning curve.

**Figure 1:**
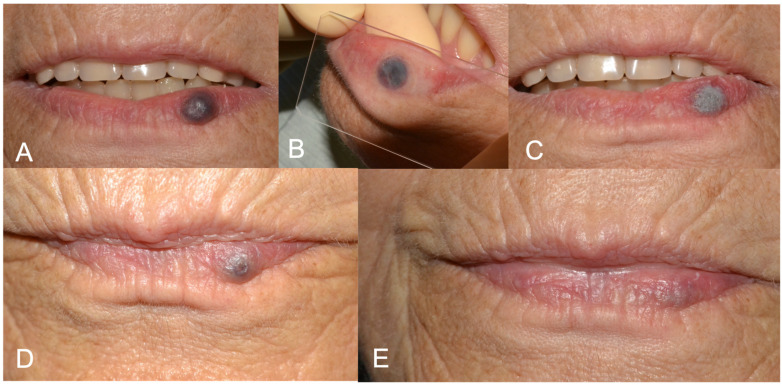
(**A**) Preoperative aspect of lower lip vascular malformation (VM); (**B**) glass slide compression before laser forced dehydration (LFD); (**C**) LFD; (**D**) 3-week follow-up showing advanced healing. A second protocol of LFD was performed in the same session. (**E**) 6-months follow up showing complete healing.

The pulsed modality was activated via a pedal, and the tip was moved after each laser shot (1 s). A net-like path was created on the lesion, and the dehydration was witnessed by the whitish lesion from the initial bluish-red color (leopard technique). This also determined the duration of the intervention, which ended when all the lesion was visually white. Eventually, the margin of the lesion was retraced to coagulate accessory vessels. More or less, the duration of the intervention was around 1–2 min (Figure 1).

### 2.3. Study Protocol: Follow-Up

Each patient was followed-up after three weeks, six months and one year, and the following aspects were evaluated:

- Pain: a Numeric Rating Scale (NRS) was used, a unidimensional 11-point scale that is used to estimate the intensity of pain in adult collaborative subjects. Values vary from 0 (=no pain) to 10 (=most severe pain ever experienced) and were reported for the whole three weeks of follow-up (the patients were asked to keep a diary). The patients were also asked to report the need to take painkillers during the day of the intervention and during the three-week follow-up;
- Bleeding: yes or no answer, considering the day of the intervention and the follow-up period;
- Scar formation: evaluated clinically at three weeks, six months and one year;
- Retreatment: at the three-week follow-up, a second LFD protocol was performed, if necessary, which, in turn, was followed by a further three-week follow-up.

## 3. Results

A total of 30 patients were included in the present study; 16 were females (53%) and 14 males (47%). The median age was 67 years old (minimum 41, maximum 86), 90% of the patients had a positive past and present medical history and 50% of patients took at least three types of medications. Further, 15 patients (50%) suffered from hypertension (under treatment) and/or previous/actual cardiac disease; 14% of patients (47%) declared hypercholesterolemia or dyslipidemia; and 17% of patients suffered from diabetes. The sample also included 10 oncological patients (previous or actual). Four patients (13%) were allergic to penicillin or NSAIDs (non-steroidal anti-inflammatory drugs). To note, six patients were under antiaggregant or anticoagulant treatment. Most patients (22) were non-smokers. Table 1 reports the demographic data.

Lesions were between 2 and 25 mm wide (mean dimension 8.3 mm). Additionally, 4 patients were affected by tongue lesions, 2 patients were affected by gingival lesions, 19 patients were affected by lip lesions, 1 patient was affected by palatal lesion and 4 patients were affected by cheek lesions. The intervention was performed for repeated bleeding in 13 patients, for aesthetic concern in 11 patients and for cluttering sensation in 6 patients.

All the patients obtained a complete disappearance of lesions after LFD, irrespective of the lesion’s dimension and site. Only one patient required a second LFD protocol at the three-week follow-up (the patient with the biggest lesion). Only one patient experienced a slight pain (NRS = 4) on the same day as the intervention, not taking any painkillers, whereas two declared a slight tingling immediately after the procedure. The NRS was 0 for all the patients the day after the intervention and for the whole duration of the follow-up. None of the patients took painkillers the day of the intervention and during the follow-up. Only one patient (the one under anticoagulants) declared slight bleeding the day of the intervention, which she easily managed via gauze compression at home. One patient showed a small non-retracting and non-painful scar at the three-week follow-up. No recurrences were found at the six-month and one-year follow-up. One patient showed ulcer formation three weeks after the LFD procedure. This happened on the gingiva, a site where slide compression is more challenging, and may be due to excessive heating. Nonetheless, ulceration was asymptomatic and ended in restitution ad integrum. Table 2 reports detailed characteristics of the interventions.

**Figure 2:**
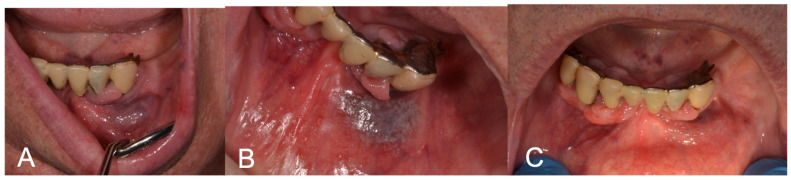
(**A**) Preoperative aspect of gingiva and lip VM; (**B**) LFD; (**C**) 3-week follow-up showing complete healing after LFD.

**Figure 3:**
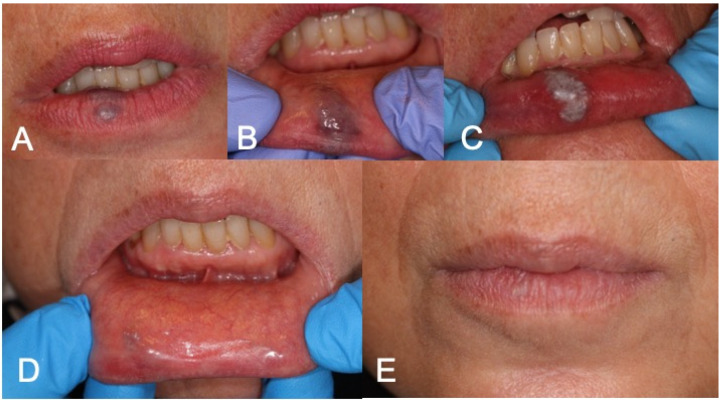
(**A**,**B**) Preoperative aspect of lower lip VM; (**C**) LFD; (**D**,**E**) 3-week follow-up complete healing after LFD.

Figure 1, Figure 2 and Figure 3 show clinical cases where LFD was applied.

## 4. Discussion

Vascular anomalies are commonly encountered in the head and neck region and are classified as hemangiomas and VM. While their pathogenesis is still unclear, they frequently require medical treatment or surgical removal. Selection of the most appropriate treatment modality is still a debated theme [9]. Considering benign lesions, treatment modalities include surgical excision, systemic corticosteroids, embolization, cryotherapy, interferon-α, radiation and sclerotherapy [10].

In general, systemic corticosteroids and interferon-α are eligible for large lesions, especially congenital hemangiomas, whereas embolization is suitable for large VMs. Local and conservative approaches are dedicated to small lesions, especially intralesional corticosteroids, cryotherapy, sclerotherapy and surgery [10,11]. The advent of laser therapy in various fields of oral medicine made it a further alternative, especially with conservative non-contact modalities like photocoagulation and LFD [12]. Multiple devices have been proposed over time, including semiconductors, 514 nm argon, 532 nm KTP (potassium titanyl phosphate), 585 nm FPDL (flash lamp-pumped pulsed dye laser), 755 nm alexandrite, 810–940 nm diode, 1064 nm Nd:YAG (neodymium-doped yttrium aluminum garnet), p-NdYAG and 10,600 nm CO2 (carbon dioxide) lasers [13,14].

One of the most tested is the Nd:YAG laser, characterized by deep penetration and high hemoglobin absorption but influenced by an important thermal reaction that leads to apoptosis and cellular death. Necrosis, scarring and infection are the main reported complications, despite the treatment being overall well tolerated and painless. Some authors suggest to cool the lesion down with water during laser enlightening [12]. In our study, we did not cool the lesion down using water but interposed a glass slide between the lesion and the laser beam, in order to reduce the risk of overheating and, concomitantly, the risk of perforation and bleeding. This technique makes LFD predictable, even for inexpert operators. Some patients may complain about slight discomfort when enlightening the lesion, and anesthesia is not recommended since the swelling induced by the liquid injection may limit the penetration of the beam. For this reason, it may be useful to blow air or keep the suction pump close to the lesion while the laser is in action.

In our study, we employed a multiwavelength diode laser. The 810–830 nm diode lasers are selectively adsorbed by hemoglobin and poorly adsorbed by water, allowing for deep penetration into tissue (around 4–5 mm) and favoring a photocoagulative process, generated by heat, up to a depth of 7–10 mm [15]. The diode lasers are usually equipped with a manageable optic fiber kept at a regular 2–3 mm distance from the lesion but not held too long while in action in order to avoid superficial damage and bleeding. The exposure is described to vary between 5 and 10 s, with an energy output of 2.5–6 W. Photocoagulation should extend a bit beyond the visible lesion in order to avoid sloughing and hemorrhage. The preferred modality is usually continuous wave [16]. In our treatments, the blue wavelength was chosen because of its greater specificity for hemoglobin and the infrared wavelength to ensure a sufficiently deep penetration into the tissue. In addition, we did not employ a continuous-wave modality but activated the beam with short impulses via a pedal, limiting the accumulation of heat into the tissue, following the concept of multiple-spot irradiation. The concept of multiple-spot irradiation (also called the “leopard technique”) was proposed more than 40 years ago, with the idea of sparing the epithelium by separating irradiation spots. In bigger lesions, part of the epithelium is left untreated, and, once the epithelium has matured and stabilized, the remaining areas can be treated, while the previously treated areas have already healed [8]. Other authors have proposed a continuous-wave modality, but this led to ulcerations in all treated patients, frequently accompanied by the need to take multiple painkillers [8]. Working progressively in some parts of the lesion allows the operator to visually understand if the technique is working, since the irradiated area turns from bluish red to white, and lets the operator decide whether to go on or treat the remaining area in a following appointment, in order to reduce the risk of perforation, overheating and ulceration. At the same time, it is very important to extend photocoagulation while allowing a safety margin that extends slightly beyond the visible lesion. This increases the rate of success and reduces tissue sloughing [16]. In our cohort, we treated quite small lesions, and except for one that required a second session, all patients were completely healed after the first follow-up. A non-neglectable result is the complete absence of pain and need for analgesics after the procedure.

Another risk of this “blind” technique is that you do not exactly know the deepness of the lesions, and LFD may be ineffective in very deep lesions. Despite some authors having demonstrated that large hemangiomas often stop growing after laser irradiation, others have demonstrated that deeper lesions are not treatable [14,17]. In our study, we treated small lesions, and we can affirm that the rate of success is very high with simple VMs, which are the most frequent in the oral cavity. Moreover, as LFD is very conservative, we believe it is worth proposing as a first-line technique, leaving more invasive approaches like intralesional coagulation and excision to failed treatments [18]. At times, LFD can be considered a tool of compromise when lesions are complex and risky, maybe with the aim of reducing their dimension and cluttering. As an alternative, LFD can be applied to reduce the dimension of the lesions, and then the operator may decide to proceed or not with a second step [18]. In complex cases, including vascular tumors, high-flow lesions, combined lesions, lesions of major named vessels and lesions associated with other anomalies [1], a more detailed diagnosis via magnetic resonance imaging is mandatory before the clinical procedure, especially in big lesions, to avoid the risk of unwanted complications, and, potentially, ultrasound-guided intervention should be considered [19,20]. Obviously, LFD should not be applied when there is suspicion of a malignant condition, since histopathological examination is not possible.

Ultimately, most of our patients suffered from general health conditions, and most of them took one or more medications, including an antiaggregant and anticoagulants. As confirmed by other authors, the risk of bleeding is so minimized that drug interruption is not required, even in the case of medications that increase the risk of bleeding [2]. In addition, diabetic patients may show delayed healing in the case of surgical interventions, and LFD nullifies this risk. The biostimulation offered by the combined wavelengths enhances tissue regeneration and wound healing, even in rare cases of superficial ulceration. In our cohort of patients, even including a certain percentage of diabetic subjects, we had no complications.

## 5. Conclusions

LFD is very effective and painless. The technique can be performed without local anesthesia, limits the duration of the intervention and is completely atraumatic. Interposing a glass slide, when feasible, drastically reduces the risk of bleeding, but, also, in cases of anatomical obstacles to slide compression (hard palate, gingiva, floor of the mouth), the multiple-spot technique can be applied, reducing the power based on a visual whitening of the lesion and performing multiple sessions until complete resolution of the pathology. The absence of pain during laser application and from the day after the intervention until the completion of follow-up is another point in favor of conservative techniques, considering that most approaches require the use of painkillers after the procedure [21]. Despite the number of patients we included in the study being limited, this technique seems promising and deserves further investigation.

## Figures and Tables

**Table 1:** Medical history, medications, smoking habit (for ex-smokers: n (number of years since they quit smoking)). Abbreviations: NSAIDs: non-steroidal anti-inflammatory drugs; AMI: acute myocardial infarction; GERD: gastroesophageal reflux disease; OCBP: obstructive chronic broncopneumopathy; Ex (month/year): ex-smoker (months/years from when he/she quit smoking).

ID	Gender	Age	Medical History	Medications	Smoking (n)
1	F	63	Carotid stenosis, hypertension, hypercolesterolemia, allergic to preservatives and fragrances	Antiaggregants, anticholesterolemics, anti-hypertensive	No
2	M	66	Myelodisplasia (2011), hypertension, prostate cancer (2015)	Xantin oxidase inhibitor, anti-hypertensive, anticholesterolemics	Ex (18 years)
3	M	76	Hypertension, hypercolesterolemia	Anti-hypertensive, anticholesterolemics, antiaggregants	No
4	M	76	Hypertension, discal hernia (intervention in 2017)	Anti-hypertensives	No
5	M	69	Allergic to penicillin	No	No
6	M	60	GERD	Proton pump inhibitor	No
7	F	59	Uterus cancer (2016), allergic to penicillin	Levotiroxine, benzodiazepine.	Ex (7 months)
8	F	67	Heart failure, hypertension, dyslipidemia, polyarhtrosis, osteoporosis, lung cancer (in 2000 subdued to surgical intervention and chemo-radiotherapy), thyroid nodules, hepatomegaly, AMI in 2002. Pacemaker since 2004, OCBP. Allergic to penicillin, NSAIDS, fluoroquinolones and triciclic antidepressants.	Anticoagulants (Coumadin), bronchodilator, anti-hypertensives, diuretics, benzodiazepines, Proton pump inhibitor, digossin, anticholesterolemics, antilipidemics, antidepressants	No
9	M	41	None	No	Yes (10/die for 20 years)
10	F	67	Autoimmune hypothiroidism, breast cancer	Iodium	No
11	F	67	Autoimmune hypothiroidism	Iodium	No
12	F	66	None	None	Yes (10)
13	M	75	None	None	No
14	F	66	Osteoporosis	Colecalciferol	No
15	F	69	Thyroid Cancer, Hypertension, hypercolesterolemia	Levothyroxine, Anti-hypertensives Anticholesterolemics	No
16	F	69	Thyroid Cancer, hypertension, hypercolesterolemia	Levothyroxine, anti-hypertensives anticholesterolemics	No
17	M	70	Diabetes, hypercolesterolemia, gout, prostate cancer (2018)	Proton Pump Inhibitor, anti-hypertensive, Antiaggregants, Oral antidiabetics, Finasteride	No
18	M	70	Diabetes, hypercolesterolemia, gout, Prostate Cancer (2018)	Proton pump inhibitor, anti-hypertensive, antiaggregants, oral antidiabetics, finasteride	No
19	M	86	Benign prostate hypertrophia, chronic renal failure, atopic dermatitis eczema, hypertension.	Silodosin, anti-hypertensives, beta-blockers	No
20	M	67	Diabetes, Parkinson’s disease, hypertension, hypercolesterolemia	Antidiabetics, diuretics, selegilin	Yes (20)
21	M	67	Diabetes, Parkinson’s disease, hypertension, hypercolesterolemia	Antidiabetics, diuretics, selegilin	Yes (20)
22	M	80	Myocardial infarction (2018)	Antiaggregant, statins, collyrium	No
23	F	63	Thyroid cancer, hypertension, hypercolesterolemia	Levotiroxine	No
24	F	62	Melanoma, allergic to cats	None	No (ex 10 years)
25	F		Lung cancer, diabetes, syderopenia, vasculopathy with vertebral collapse	Antidiabetics, Proton pump inhibitors, bronchidilator, steroids, Dibase, oxygen therapy.	No
26	F	78	Hypertension	NSAID, anti-hypertensive, diuretic	Ex (18)
27	F	59	Hypothyroidism, Hypertension	Levotiroxin, anti-hypertensives, diuretic	No
28	F	74	Gouge, hypercolesterolemia, allergic to penicillin	Allopurinol, anticholesterolemics	No
29	M	73	Hypertension, ischemic stroke, hyperlipidemia, osteoporosis	Antiaggregants, antilipidemics, anti-hypertensives, benzodiazepines, colecalciferol	No
30	F	71	Ipercolesterolemica, emicarnica	Anticholesterolemics, triciclinc antidepressants, beta blockers	No

**Table 2:** Characteristics of lesions, intervention and outcomes. Abbreviations: LFD: laser forced dehydration.

ID	Site	Reason for Intervention	Dimension (mm)	Number of LFD Sessions	Pain	Bleeding	Scar	Side Effects
1	Lower Lip	Aesthetic	8	1	No	No	No	
2	Left Cheek	Bleeding	10	1	No	No	No	Slight tingling immediately after application
3	Lower Lip	Bleeding	3	1	No	No	No	
4	Left Cheek	Bleeding	15	1	No	No	No	
5	Tongue	Clutter	11	1	No	No	No	
6	Tongue	Clutter	6	1	Yes	No	Yes	Slight visible scar, without pain or retraction
7	Left Cheek	Bleeding	12	1	No	No	No	
8	Lower Lip	Bleeding	20	1	No	SI	No	Slight bleeding the day of the intervention
9	Left Cheek	Bleeding	8	1	No	No	No	
10	Lower Lip	Aesthetic	8	1	No	No	No	
11	Lower Lip	Clutter	25	2	No	No	No	
12	Lower Lip	Aesthetic	3	1	No	No	No	
13	Tongue dorsum	Bleeding	3	1	No	No	No	Slight tingling the day of the intervention
14	Lower Lip	Aesthetic	10	1	No	No	No	
15	Lower Lip	Aesthetic	10	1	No	No	No	
16	Lower Lip	Aesthetic	5	1	No	No	No	
17	Gingiva	Clutter	15	1	No	No	No	
18	Lower Lip	Bleeding	10	1	No	No	No	
19	Lower Lip	Aesthetic	5	1	No	No	No	
20	Lower Lip	Aesthetic	2	1	No	No	No	
21	Upper Lip	Aesthetic	5	1	No	No	No	
22	Gingiva	Bleeding	15	1	No	No	No	Ulcer after treatment
23	Lower Lip	Aesthetic	4	1	No	No	No	
24	Lower Lip	Aesthetic	7	1	No	No	No	
25	Palate	Bleeding	8	1	No	No	SI	
26	Lower Lip	Bleeding	6	1	No	No	No	
27	Tongue Dorsum	Clutter	3	1	No	No	No	
28	Lower Lip	Bleeding	6	1	No	No	No	
29	Lower Lip	Bleeding	3	1	No	No	No	
30	Lower Lip	Clutter	4	1	No	No	No	

## Data Availability

The original contributions presented in the study are included in the article, further inquiries can be directed to the corresponding author.
